# Accidental Drowning: The Importance of Early Measures of Resuscitation for a Successful Outcome

**DOI:** 10.1155/2018/7525313

**Published:** 2018-06-04

**Authors:** Ljiljana S. Sulovic, Aleksandar P. Pavlovic, Jovan B. Zivkovic, Zorica N. Zivkovic, Snezana S. Filipovic-Danic, Slađana V. Trpkovic

**Affiliations:** ^1^Medical Faculty, University of Pristina in Kosovska Mitrovica, Children's Hospital, Kosovska Mitrovica, Serbia; ^2^Medical Faculty, University of Pristina in Kosovska Mitrovica, Surgery Clinic, Department of Intensive Care, Kosovska Mitrovica, Serbia; ^3^Medical Faculty, University of Pristina in Kosovska Mitrovica, Neurological Clinic, Kosovska Mitrovica, Serbia

## Abstract

**Case Report:**

The case of a drowning teenager is described involving application of cardiopulmonary resuscitation (CPR) by an untrained rescuer in the field and fast transport to a hospital enabling a positive resuscitation outcome despite an underorganized emergency medical service in a rural area. In our case hypoxia led to extended functional disorders of the cardiovascular system, which fully recovered after adequate therapy.

**Conclusion:**

Knowledge about BLS measures by ordinary citizens, together with continuous education of health professionals concerning modern techniques of CPR, is crucial for increasing the number of patients surviving after cardiac arrest.

## 1. Introduction

“Drowning is the process of experiencing respiratory impairment from submersion/immersion in liquid” [1]. It is the third leading cause of mortality due to unintentional injury, accounting for 7% of all injury-related deaths [2]. The World Health Organization (WHO) states that every hour more than 40 people die from drowning, which is 372,000 deaths per year [3]. The etiology of drowning is multifactorial and varies with age and geographic location [4]. The incidence of such cases in Europe differs from country to country and is the largest in Eastern Europe [5]. The incidence among men is more than double that for women (in the US 4.6: 1, in Europe 4.2: 1). Probable reasons for this are greater sporting activity and risky male behavior (abuse of alcohol or drugs) [6]. There are no official data in Serbia, but according to the reports from the field, there are between 120 and 150 victims of drowning. Most affected groups are children under five years old and then the boys from 15 to 19 years old.

Many factors affect drowning, including those of sociodemographic origin [7]. According to Cummings and Quan more than one-third of drowned individuals had a measurable blood alcohol concentration [8]. Some chronic diseases and conditions, such as cardiac rhythm disorders, hypertension, diabetes mellitus, anxiety, autism or acute conditions like myocardial infarction, or epileptic seizures, are also risk factors for drowning [9].

## 2. Case Report

A sixteen-year-old boy nonswimmer was hospitalized after drowning in cold river water. Based on data given by the passing eyewitness, the teenager jumped into the river to retrieve a sheep he was tending but immediately disappeared from the water surface. This youth of the same age promptly dived into the river, found the victim's body on the bottom at a depth of 2 metres, raised him, and swam to the shore, which was about 3 metres away. Based on the rescuer's statement, the drowning boy was unconscious, was not breathing, had blue lips and nail pates, and had very cold skin. Due to knowledge about First Aid acquired in high school, the rescuer started measures for basic life support (BLS (Basic Life Support)), freed the airway, performed several mouth to mouth breaths, and started chest compression. After 2-3 minutes the victim vomited, threw up water, coughed, and started breathing. In the next few minutes he regained consciousness. In the meantime, members of his family had been called and they brought the patient to our institution in a private vehicle.

At arrival he was somnolent, oriented, anxious, and pale, with tachypnea (number of respirations 30/min), with hypothermic-body temperature 35.1°C, shivering (whole body), and with the subjective sensation of cold. He was admitted to the JIT and the following was monitored: ECG TA, pulse, body temperature, and pulse oximetry. A nasogastric tube and a urinary catheter were placed. Auscultation of both lungs revealed weakened breathing sounds, especially in the basal and middle parts, with many early and late inspirium cracklings and low tone whistling. Cardiac action was arrhythmic accompanied by tachycardia up to 120/min, BP (blood pressure) 100/55 mmHg. There were no signs of neurological deficit or lateralization, GCS (Glasgow Coma Scale) 13/15.

Using transcutaneous pulse oximetry at admission we measured the following: O_2_ saturation, which was 80% (during the course of treatment it normalized); pH 7.32; O_2_ 54.8 mm Hg; CO_2_ 55 mm Hg; BE—1.60 mmol/L; C-reacting protein 96 mg/L; Er 5.11 × 10^12^/L; Hb 136 g/L; Le 14.3 × 10^9^/L; glycemia 4.0 mmol/L. Chest radiography showed spotty shadows in the middle and inferior lobes with free costodiaphragmatic recess sinuses bilaterally.

The ECG at arrival indicated an irregular rhythm, HR (heart rate) 120/min with short-term atrial fibrillation that later corrected spontaneously into normal sinus rhythm.

Immediately upon admission we continued reanimation. The patient was warmed up with thermal blankets. Oxygen therapy was applied using an oxygen mask with reservoir without rebreathing, at a flow rate of 8 l/min, decreasing to 6 l/min in order to maintain SpO_2_ ≥ 94%. Parenteral antibiotic therapy (III generation cephalosporin, aminoglycosides, and metronidazole) and parenteral rehydration were administered.

On the fifth day after admission the patient showed complete recovery, including withdrawal of the chest radiographic symptoms and no pathological signs were visible on physical examination of the lungs.

However, during each attempt to get up, we registered atrial fibrillation or individual SVES (supraventricular extrasystole) and VES (ventricular extrasystole) triplets. On echocardiographic examination the structure of the left ventricular myocardium was hyperechogenic; echocardiography was performed with slightly restricted LVEF

During halter ECG examination we registered multiple strings of VES triplets, frequency 150 per minute. An appropriate dose of Presolol was included in the therapy, with advice of strict inaction and avoidance of any kind of physical exertion.

Two months later the control echocardiography revealed that heart function had improved further, FS 0.34%; EF > 45%.

## 3. Discussion

Knowledge of the techniques of BLS should become part of the general education of modern man. The exact measures carried out by the eyewitness at the scene of the accident were crucial for survival of the victim and reduction of neurological sequelae in the period after cardiac arrest. Early initiation of BLS measures increases the chances of survival up to 2–4 times [9–11].

The average time from an emergency service call to the arrival of an ambulance at the place of an accident is usually 5–8 minutes or 8–11 minutes before giving the first shock (defibrillation) [10, 12]. During this period the victim is completely dependent only on lifeguards or companions in the field. In our case, CPR was performed in a rural area, without a well-developed connection with EMS (Emergency Medical Services) and the victim was left to the knowledge and skills of a passer-by and family members who arranged transport to the hospital.

The diagnosis of acute heart failure (cardiac arrest (CA)) should be made as soon as possible without delay. One should always bear in mind that after 3–5 minutes there is irreversible damage to brain cells after which each resuscitation is less successful because of progressive brain death [10]. Therefore, the so called “arrest time” is very important, that is, the time from the occurrence of CA to the moment of starting CPR, in which eyewitnesses play a crucial role [9]. When the diagnosis of CA is made by an untrained rescuer, the indication for starting CPR is “the victim is unresponsive and not breathing normally.” No one should expect a layman to make a diagnosis of CA based on four certain signs (loss of consciousness, cessation of breathing, pulse loss of large blood vessels, discoloration of the skin, and visible mucous membranes) [12].

ERC (European Resuscitation Council) 2015 guidelines stress the importance of coordinated communication between dispatchers of EMS and rescuers on the ground, which is reflected in rapid diagnosis of CA, dispatcher-assisted “phone CPR,” as well as location and delivery of the nearest automatic external defibrillator (AED). The EMS dispatcher can instruct to an untrained rescuer in the field to provide CPR chest compression only, except in cases when the cause of CA is asphyxia, that is, in drowned subjects and small children, where CPR is performed by a combination of mouth to mouth respiration and chest compression [10].

The process of rescuing a drowning victim involves a quick and careful way of pulling the person out of the water, recognition of the need to call the EMS, giving basic life support (BLS), early defibrillation, and providing advanced life support (ALS) with emergency transport to a hospital [13–15]. In our case, the knowledge about and quick initiation of BLS measures by the rescuer were of crucial importance in saving the life of the drowning boy.

Primary and secondary hypothermia can occur after rescue from drowning. Primary hypothermia happens suddenly, following submergence in cold water (lower than 5°C) and this provides some kind of protection against hypoxia (a neuroprotective effect especially in children) [15]. Secondary hypothermia occurs during measures of reanimation and there is no protective effect [9, 12]. We eliminated this in our patient by simple wrapping up in warm blankets and infusions heated to room temperature.

In our patient, drowning and prolonged hypoxia led to complications in the cardiovascular system. The dilemma of the physician treating him was whether the cardiac symptoms were the consequence of drowning or had they existed earlier. In the medical history given by the patient and family members there was no information about any cardiovascular problems in the period before the accident, so the findings for the heart were defined as “de novo” and caused by prolonged hypoxia during drowning.

Lack of oxygen in parts of the heart creates small hypoxic fields which lead to locally formed arrhythmias that can cause CA. The cells of the conduction system are more sensitive to hypoxia than myocardial cells. Cardiac dysfunction can also occur as a result of hypoxia associated with strangulation. Reviewing the literature we obtained data on the initial acute effects of drowning (hypoxia and hypothermia) on the cardiovascular system but not about extended, long-term after-effects as described here [16].

Changes in the heart muscle due to high systemic and pulmonary vascular resistance may persist even after the establishment of adequate oxygenation, ventilation, and perfusion. Pathological alterations in the lungs may occur by several pathophysiological mechanisms, including increased resistance of peripheral airways, different degrees of laryngospasm, reflex vasoconstriction of the pulmonary arteries leading to pulmonary hypertension, decrease of ventilation-perfusion ratio, aspirated water, loss of surfactant, anatomical changes in alveolar epithelial cells, and others [14, 17].

During the postarrest period in the next 72 hours after rescue from drowning there is a high risk of developing ARDS. A protective ventilatory strategy, using PEEP (positive end-expiratory pressure), is beneficial in that interval, because it leads to reopening of collapsed alveoli. Pneumonia often occurs in these patients during the postarrest period, especially after drowning in polluted water. The application of broad spectrum antibiotics is recommended [9, 14]. The state of our patient did not require tracheal intubation or mechanical ventilation. Oxygenation with 100% O_2_ via an oxygen mask led to satisfactory saturation. In such patients the goal is to achieve SpO_2_ 94–98% [14].

The neurological outcome, especially serious permanent neurological damage, primarily depends on the length of hypoxia. Teaching BLS in high school significantly raises the number of people able to perform BLS; thus the awareness of the population of the importance of early BLS is raised. In Serbia teaching and studying BLS is standard in education.

## 4. Conclusion

Early use of BLS by a rescuer on site is crucial for the survival of victims of drowning with minimal neurological sequelae in the postarrest period. When a person drowns in cold water, particularly children, the “arrest time” may be extended due to the protective effect of hypothermia. This slows metabolism and thus reduces the oxygen needs of brain cells. In our case hypoxia was followed by extended functional disorders of the cardiovascular system which later recovered fully after suitable therapy. Knowledge of BLS measures by ordinary citizens as well as continuous education of health professionals regarding modern techniques of CPR is crucial for increasing the number of patients surviving after CA.
